# What About Older Persons in Palestine? A Call for Recognition, Rehumanising and Solidarity in Gerontology

**DOI:** 10.1093/geroni/igaf122.2671

**Published:** 2025-12-31

**Authors:** Yudi Pamela, Suero Samboy, Preetha Joseph

**Affiliations:** Free University of Brussels (VUB), Society and Ageing Research Lab, Brussels, Brussels Hoofdstedelijk Gewest, Belgium; University of Galway, Galway, Galway, Ireland

## Abstract

While the impact of humanitarian crises caused by armed violence is well-documented, the specific experiences of older persons remain largely overlooked, particularly within academia and the field of gerontology. Amidst the recent genocide in Gaza, older Palestinians represent a stark example of this silence and systemic neglect. After decades of oppression under occupation, their plight is now intensified by high mortality risk, forced displacement, collapsed essential services and devastation of their communities. Our work argues that the experience of older Palestinians must serve as a wake-up call for gerontology and international policy agendas, as it exposes a failure to uphold global commitments to healthy ageing and human rights protection. Structural dispossession, exile, disrupted care and support networks, and destruction of living environments are severely undermining older Palestinians’ ability to age in place or experience age-friendly conditions. Beyond reflecting on the crisis in Gaza, we critique the broader neglect of older persons hit by armed violence. We urge gerontologists to adopt an emancipatory social science approach through acts of recognition, rehumanisation, and solidarity towards older Palestinians, within teaching, research, dissemination, and scholar-activism. Against the backdrop of uncertainty following the ceasefire announced in January 2025, gerontology can play a vital role in ensuring that older persons are not left behind in efforts to rebuild a Palestine free from occupation. Breaking the silence on Palestine can reaffirm gerontology’s moral purpose and legitimacy as a value-based discipline, and contribute to advancing a much-needed transformative agenda to address injustices affecting older persons around the world.

